# Adverse perinatal outcomes in borderline amniotic fluid index

**Published:** 2016-11

**Authors:** Ashraf Jamal, Maryam Kazemi, Vajiheh Marsoosi, Laleh Eslamian

**Affiliations:** *Department of Obstetrics and Gynecology, Shariati Hospital, Tehran University of Medical Sciences, Tehran, Iran.*

**Keywords:** *Perinatal Outcomes*, *Pregnancy*, *Borderline Amniotic Fluid Index*

## Abstract

**Background:**

Normal amniotic fluid predicts normal placental function, fetal growth and fetal well-being.

**Objective:**

To determine adverse pregnancy outcomes in borderline amniotic fluid index (AFI).

**Materials and Methods:**

Pregnant women (37-40 wks) with diagnosis of borderline AFI between December 2012 and August 2014 were identified. Antepartum, intrapartum and neonatal data were collected and compared with those of pregnant women with normal AFI. An AFI less than 8 and more than 5 cm was defined for borderline AFI. Pregnancy outcomes included Cesarean section for non-reassuring fetal heart rate, meconium stained amniotic fluid, 5-min Apgar score <7, low birth weight, umbilical cord blood pH at term and NICU admission.

**Results:**

Gestational age at delivery in pregnancies with borderline AFI was significantly lower than normal AFI. Cesarean section rate for non-reassuring fetal heart rate in women of borderline AFI was significantly higher and there was an increased incidence of birth weight less than 10^th^ percentile for gestation age in borderline AFI group. Incidence of low Apgar score and low umbilical artery pH in pregnancies with borderline AFI was significantly higher than women with normal AFI. There were no significant difference in the rate of NICU admission and meconium staining in both groups.

**Conclusion:**

There are significant differences for adverse pregnancy outcomes , such as Cesarean section due to non-reassuring fetal heart rate, birth weight less than 10^th^ percentile for gestation age, low 5 min Apgar score and low umbilical artery pH between pregnancies with borderline and normal AFI.

## Introduction

Amniotic fluid is indicator of normal placental function and is the most important component of fetal well-being tests. Amniotic fluid index (AFI) is the preferred method of amniotic fluid measurement in pregnancy although single deepest pocket is used in pregnancies suspected to oligohydramnios. For measuring AFI the uterus divided into four equal quadrants. AFI is the sum of deepest pocket from each quadrant. The normal range of AFI is between 5-24 cm while any value above 24 cm will be considered as Hydraminios and with value below 5 cm will be indicated as Oligohydraminios. Borderline AFI that is defined 8> AFI >5 is challenging in obstetrics for association with adverse pregnancy outcomes. 

Although different ranges have been suggested for its definition, the majority of studies have used the range between 5 and 8 cm. Some studies have considered definition between 5 and 10 cm and still some have used the percentile 2.5-5 (1-3). It seems that more accepted range for borderline AFI is between 5 and 8 cm (4, 6). Regarding potential risks associated with borderline amniotic fluid, results are contradictory. Asgharnia *et al* reported increased incidence of preterm delivery, labor induction, low Apgar score, Intra Uterine Growth Restriction (IUGR) and Neonatal Intensive-Care Unit (NICU) admission in pregnancies of borderline amniotic fluid while Choi *et al* in a recent study did not reach this conclusion (7, 8). 

Gumus and colleagues also reported higher incidence of preterm delivery, fetal distress and birth weight less than 10^th^ percentile in borderline AFI (9). Based on variation in adverse pregnancy outcomes associated with borderline amniotic fluid, it seems that there is still need for more evidence to suggest antepartum fetal assessment in these pregnancies. To clarify the issue, we conducted a comparative study between two groups of pregnancies with borderline and normal amniotic fluid.

## Materials and methods

This cross-sectional study was performed on pregnant women of 37-40 wks of gestational age with borderline AFI who delivered in Shariati Hospital within one week after measuring AFI. Borderline AFI was defined 5< AFI <8. Ultrasonographic examinations for measuring AFI based on standard four quadrants protocol were performed in perinatology division, using the Antares (Siemens, Germany) with a 3.5 MHz curvilinear transducer by different perinatologists. Umbilical artery pH was measured immediately after delivery. Control group was low risk pregnant women (37-40 wks) with normal AFI who delivered in the same hospital during a week after measuring AFI. AFI between 8 and 24 cm was considered as normal AFI. Informed consent was taken from both groups and the study protocol was approved by the Ethic Committee of Tehran University of Medical Sciences. 

Inclusion criteria were term, singleton pregnancy (37-40 wks), borderline or normal AFI, intact amnion membrane, cephalic presentation, no fetal anomalies, no maternal medical disease, no contradiction for normal vaginal delivery (macrosomia, non-vertex fetal presentation, placenta previa, history of cesarean section) and delivery during a week after AFI measurement. Exclusion criteria were those gestational ages less than 37 wk or more than 40 wk, rupture of membrane, fetal anomalies, maternal medical disease and illicit drug users. Data was extracted from their medical records and was inserted into the checklist. Frequency of adverse perinatal outcomes such as Cesarean section due to non-reassuring fetal heart rate, meconium staining, birth weight less than 10^th^ percentile for gestation age, low 5 min Apgar score, low umbilical artery pH and NICU admission were compared within groups.

**Statistical analysis**

Qualitative variables were presented with number and percentages and quantitative variables were presented with mean and standard deviation. Study data were analyzed with independent samples t-test for quantitative and ^2^ test for qualitative variables. Statistical level of confidence was 95% and all p˂0.05 were assumed as significant results.

## Results

There was no significant differences between maternal age in normal and borderline AFI groups (26.6±5.2 vs. 25.9±5.2, respectively, p=0.53). Mean of gestational age at delivery in women with borderline AFI (37 wk and 5 days) was significantly lower than women with normal AFI (38 wk and 6 days) (p<0.001). Rate of Cesarean section among pregnant women with borderline AFI was significantly higher than pregnant women with normal AFI (60.9% vs. 40.6%, respectively, p=0.02). Frequency of Cesarean section due to non-reassuring fetal heart rate was significantly higher in borderline AFI group compared to normal AFI (43.6% vs. 28.7%, respectively, p=0.038). Variable deceleration rate among pregnant women with borderline AFI was significantly higher than pregnant women with normal AFI (p=0.002).

There was no significant differences for meconium staining between normal and borderline AFI groups (14.1% vs. 17.2%, respectively, p=0.63). Mean birth weight in borderline AFI pregnancies was significantly lower than group with normal AFI (2853.9±240.5 gr vs. 3195.3±394.3 gr, respectively, p<0.001) and frequency of birth weight less than 10^th^ percentile was significantly higher among women with borderline AFI in comparison with women with normal AFI (18.7% vs. 9.4%, respectively, p=0.034). Mean of 5 min Apgar score was significantly higher among normal AFI group in comparison with borderline AFI group (9 vs. 8.5, respectively, p<0.001). But frequency of 5-min Apgar score <7 did not have significant differences between normal and borderline AFI groups (6.2% vs. 9.6%; respectively, p=0.14). 

Mean of the umbilical artery pH in normal group was significantly higher than borderline group (7.28% vs. 7.21%, p<0.001), but there was not found difference in incidence of umbilical artery pH <7 between two groups (11.2% vs. 7.8%, respectively, p=0.38). There was not a significant difference in NICU admission between two study groups (6.8% vs. 10.5%; respectively, p=0.17). There was no case of stillbirth in both study groups (Table I).

**Table I:** Frequency of adverse perinatal outcomes between pregnancies with borderline and normal AFI

**Prenatal outcome**	**Normal AFI**	**Borderline AFI**	**p-value**
Gestational age at delivery (wks, days)	38 weeks and 6 days	37 weeks and 5 days	<0.001*
Normal vaginal delivery (%)	59.4%	39.1%	0.02*
Cesarean section (%)	40.6%	60.9%	0.02*
Cesarean due to non-reassuring fetal heart rate (%)	28.7%	43.6%	0.038*
Birth weight (gr)	3195.3 ± 394.3	2853.9 ± 240.5	<0.001*
Meconium staining (%)	14.1%	17.2%	0.63
Birth weight less than 10^th^ percentile (%)	9.4%	18.7%	0.034*
5 min Apgar score (no)	9	8.5	<0.001*
5 min Apgar score <7(%)	6.2%	9.6%	0.14
Umbilical artery pH(gr)	0.12 ± 7.28	0.14 ± 7.21	<0.001*
Umbilical artery pH <7 (%)	7.8%	11.2%	0.38
Neonatal Intensive-Care Unit (NICU) admission (%)	6.8%	10.5%	0.17

AFI: Amniotic Fluid Index

* P<0.05 is statistically significant. (n=64 each group)

## Discussion

Findings of the present study showed significant association between borderline AFI and the majority of adverse perinatal outcomes. Lower gestational age at delivery, higher rate of Cesarean section and lower birth weight in borderline AFI is a common occurrence in approximately all studies for this matter and may be explained by earlier and higher rate of intervention. This explanation does not correspond with Wood’s study that showed no difference in the rate of fetal intolerance of labor (10). Increased incidence of birth weight less than 10^th^ percentile for gestation age in borderline AFI group is explained by somehow impaired placental function and fetal growth. The differences in the incidence of low umbilical cord blood pH can be explained by differences in maternal characteristics and intrapartum factors that affect blood gas status. 

The results of present study is not consistent with Phelan *et al* who reported no significant differences for fetal distress and Apgar scores in borderline group in comparison with normal amniotic fluid (11). Jeng *et al* demonstrated similar results in measures of outcomes of meconium staining, cesarean section for non-reassuring fetal heart rate and 5 min Apgar score <7 in borderline AFI (12). While Baron *et al* reported no significant difference between meconium staining, Cesarean section for fetal distress, birth weight ˂2500 gr, 5 min Apgar score <7 and NICU admission (5). Kwon *et al* showed higher rate of perinatal outcomes such as Small for Gestational Age (SGA), Cesarean section for fetal distress, 5 min Apgar score <7 and NICU admission in borderline AFI group (13).

Gumus and colleagues reported higher rate of meconium staining that is not in agreement with the present study (9). Petrozella *et al* showed that higher rate of fetal malformations in borderline AFI, but there was not found any malformation in recent study (6). Although, Luo *et al* showed no significant differences in fetal distress and fetal mortality, but they showed more emergency Cesarean section (14). Contradictory results in different studies may be explained by variation in study design and patient selection and physician’s anxiety regarding decreased amniotic fluid. There is no consensus for fetal testing and no specific protocol for prenatal care in these pregnancies. In a review by Magan and colleagues based on uncertainty of predictive value of borderline amniotic fluid for adverse outcomes the ultrasonography was suggested for fetal growth with no additional testing (4).

## Conclusion

In conclusion, findings of present study suggest that pregnancies with borderline AFI are at the risk of adverse perinatal outcomes. According to this fact, these pregnancies should be observed carefully by frequent fetal assessment, intrapartum monitoring and neonatal care. Considering no consensus for fetal testing, timely intervention and intrapartum fetal monitoring; there is still need for more studies. Studies using color Doppler assessment of cerebroplacental ratio is valuable in this group of pregnancies.

## Note

***This article extracted from M.D.*** ***thesis. (Maryam Kazemi)***
